# Public‐Private‐Partnerships for Primary Care in India, Pakistan and Bangladesh: Lessons on Pathways and Drivers

**DOI:** 10.1002/hpm.3947

**Published:** 2025-05-16

**Authors:** Shehla Zaidi, A. Venkat Raman, Mahbub Elahi Chowdhury, Farooq Azam, Priya Balasubramanium

**Affiliations:** ^1^ Global Business School for Health University College London London UK; ^2^ Faculty of Management Studies University of Delhi Delhi India; ^3^ International Centre for Diarrheal Disease Research Dhaka Bangladesh; ^4^ The World Bank Mission Pakistan Pakistan; ^5^ Public Health Foundation India Delhi India

**Keywords:** drivers, pathways, public‐private‐partnerships, south asia, universal health

## Abstract

Formalised public‐private‐partnerships (PPPs) for primary care have proliferated in the mixed health systems of India, Pakistan and Bangladesh, managed and funded by the state. This perspective provides a process‐based understanding of pathways adopted by home‐grown PPPs and underlying drivers to identify lessons for advancement under Universal Health Coverage (UHC). PPPs have been deployed to respond to local primary care needs ranging from diagnostic screening, maternity services, management of government health centres, mobile clinics to urban primary care systems. Partnerships have evolved to include a diverse range of private partners and more purposeful arrangements, with increase in service volumes, innovations albeit less standardised quality of care. The pathway of PPP instigation, rollout and sustaining in South Asia is based on local starting points by sub‐national governments, diffusion of practice across states, common interests and shared bureaucratic coalitions. Success drivers include administrative support beyond the health sector, simplified contractual and payment systems providing operational ease and decision space, and the use of relational management and digital monitoring for resolving issues. However, PPPs are constrained by either too little accountability or excessive accountability in contract design, trust deficits between private and government, and fire‐walled PPP implementation creates disconnects from national primary care planning and regulation. Donor supported projectized PPP funding and accompanying rules of business makes PPPs implementation more cumbersome. We conclude that future attention must centrally focus on pathways and drivers to impactfully introduce, scale‐up and sustain PPPs in South Asia. Emphasis must be on pathways that build on local simplified ideation, progressive adaptation and allowing contextual diversity under a larger UHC planning architecture, as opposed to centralised one‐fit and heavily technocratic initiatives. Success drivers must feature in design of PPP initiatives. Furthermore, we contend that international donor assistance should shift from projectized support for PPPs to building public sector competencies for stewardship, private sector engagement skills as well as the more traditional performance management capacity.

**Summary**

- The South Asian experience demonstrates the central role of pathways and drivers for the initiation, continuation and scale‐up of PPPs.
- PPPs must build on local needs, grounded ideation, adaptive progression rather than centralised sophisticated one‐fit designs.
- Political‐bureaucratic support beyond the health sector, simplified contracting, decision space for private partners, recurrent funding and relational management emerge as key successive drivers from the South Asian context.
- Future support is required in providing an architecture of national primary care planning and quality regulation to PPPs while allowing local diversity in implementation.
- Future attention is required to building capacity for stewardship, private sector engagement skills as well as the more traditional performance management capacity.
- A shift in international donor support is required from short‐term projectized funding to longer‐term technical support to support a practical realist configuration of PPPs for PHC.

## 1 Introduction

**FIGURE 1:**
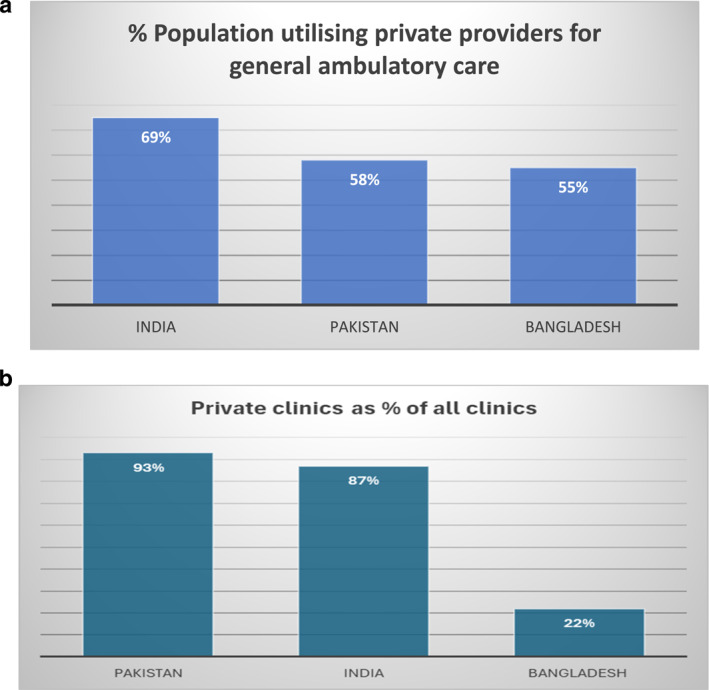
(a) % Population Utilising Formal Private Sector for General Ambulatory Care. India Private Sector Landscape in Mixed Health Systems 2020 https://www.who.int/docs/default‐source/health‐system‐governance/private‐sector‐landscape‐in‐mixed‐health‐systemsc23a2a3a‐dc7a‐4ef2‐8c11‐09d74fdb606e.pdf. Bangladesh: Private Sector Landscape in Mixed Health Systems 2020 https://www.who.int/docs/default‐source/health‐system‐governance/private‐sector‐landscape‐in‐mixed‐health‐systemsc23a2a3a‐dc7a‐4ef2‐8c11‐09d74fdb606e.pdf. Pakistan: Pakistan Social and Living Standards Measurement Survey 2019–2020 https://www.pbs.gov.pk/content/pakistan‐social‐and‐living‐standards‐measurement. * Private facilities are non‐state health facilities inclusive of both for‐profit and not‐for profit institutions. (b) % Private Clinics as a % of Total Clinics. Health Bulletin 2020 Bangladesh https://old.dghs.gov.bd/images/docs/Publicaations/Health%20Bulletin%202019%20Print%20Version%20(2)‐Final.pdf. National Health Resources Repository, 2023 India; https://cbhidghs.mohfw.gov.in/WriteReadData/l892s/Final_Central%20Bureau%20of%20Health%20Intelligene%20July%202024.pdf. WHO 2014 https://applications.emro.who.int/dsaf/EMROPUB_2014_EN_1790.pdf.

Accessible, affordable and good quality primary care services financed through public financing are essential for advancing Universal Health Coverage (UHC) in countries that rely on extensive mixed health care systems [1, 2]. Primary care delivery in Bangladesh, India, Pakistan, relies on an entrenched, growing network of private health providers who co‐exist alongside state funded health infrastructure [3] (Figure 1). These countries have a large tax‐funded infrastructure of free public sector health infrastructure but weakly functional government primary care, whereas non‐communicable diseases and rapid urbanisation put further pressure on government services contributing to reliance on private providers [4]. Private providers in these three heavily populous South Asian countries comprise of a large formal private commercial sector relying on out‐of‐pocket patient payments as well as a sizeable non‐profit sector providing subsidised servcies [5]. They offer the comparative advantage of being the frontline provider of several essential services offer more functional services better patient satisfaction [5], but global concerns related to quality of care, efficiency, value for money and for‐profit incentives of the private sector must be considered when designing partnership modalities [6]. PPPs for primary care have proliferated in Bangladesh, India and Pakistan from informal, small‐scale arrangements to more purposive, formal and substantially large‐scale arrangements. However, PPPs continue to be implemented as standalone arrangements proliferating in parallel to UHC initiatives that are centred on hospital‐based national health insurance programs, over‐looking primary care [7].

Public private partnerships (PPPs) are formal agreements between public and private partners to deliver a shared goal within a stipulated time and specify how the partnership will be governed [8]. The impetus for governments to partner with the private sector to deliver services under public sector goals originated under the New Public Management (NPM) agenda [9]. Effectively the public sector would shift from an exclusive role on service delivery to stewarding the financing and monitoring of contracted private providers so as to overcome complex public sector bureaucracies that impede efficient service delivery and harness the private sector for population health needs [9]. Theoretical guiding frameworks from well developed economies emphasise formal controls including tight well‐designed contracts, detailed performance monitoring and legal risk management as integral for impactful PPPs [10]. However, transactional mechanisms for PPPs are less developed in low‐middle‐income country (LMIC) settings where informalities and stakeholder interests play a salient role for contractual partnerships [11]. Recent global discourse on private sector spearhead by the WHO emphasises attention to trust, relationship and alignment with national policies for country governance of private sector alongside traditional purchasing and regulatory levers [12] and calls for more evidence from the global south to build the field on private sector engagement.

This perspective aims to advance a process‐based understanding of why PPPs for primary care services have been introduced and how these have fared in South Asian context.

Its value lies in identifying lessons for practical realist designs and success pathways for alignment and scale up under the present UHC momentum. We draw on experiential insights from the three large South Asian countries of Bangladesh, India, Pakistan that share a common policy and administrative ecosystem. Our focus is on formal partnerships between governments and private providers for delivering primary care services, managed and fully or partially financed by the state. The perspective is framed by three critical questions: (i) what has driven the evolution of domestically financed PPPs? (ii) what are the defining modalities of home‐grown PPPs? (iii) what enablers and barriers are encountered in delivering and sustaining PPPs? The viewpoint draws on authors insights from the three countries supplemented by a desk review that includes extensive grey unpublished literature not available in public domain, journal articles and solicitation of views of country stakeholders. We conclude with forward looking recommendations on key drivers, pathways, and building capacity to scale‐up PPPs within the evolving UHC momentum in South Asia.

## 2 PPP Trajectory and Local Agendas

### 2.1 Growth of Home‐Grown PPPs With Limited Integration Into National Planning

PPPs have significantly proliferated in Bangladesh, India and Pakistan over the last 3 decades. PPPs have importantly shifted over time from less formal arrangements involving public financing to selected medical charities to purposive formal arrangements that fund a large range of private healthcare providers. Earlier forms of PPPs comprised of grant‐in‐aid agreements with trusted philanthropic hospitals, land‐for‐bed arrangements and occasional joint investment ventures for establishing hospitals with an expectation for free hospital services. These did not have a primary health care focus nor placed an emphasis on performance and financial accountability by private partners. Primary care engagement with private providers was initially spearheaded by donor agencies in Bangladesh, India and Pakistan with the introduction of franchising, social marketing, vouchers (demand‐side financing) and case‐based payments (supply‐side financing) for priority issues such as maternal‐child health services, family planning and Tuberculosis control. However, country governments often had a marginal role limited to signing of memorandums of understanding whereas funding, design and execution was led by international NGOs. Hence despite promising examples, donor driven PPPs struggled to transition to government oversight and funding support.

A rise in several home‐grown PPP initiatives for primary care is seen over the last 2 decades across Bangladesh, India and Pakistan managed by country governments with full or partial financing from state funds [13, 14, 15]. PPPs have emerged at the sub‐national levels and range g in scale from few districts to several states. National health policies that have shifted from an exclusive public sector focus to recognising formal engagement of the private sector (Box 1). Conversely, there has been little progress in operationally translating PPP policy intent into national PHC plans and aligning with ongoing UHC initiatives. Progress is impeded by unclarity within health ministries on best suited PPP modalities and exacerbated by erosion of institutional memory due transfer of leadership as well as little attempt to consolidate lessons from local experiences. Another constraint to national planning for PPPs is that primary care has long been considered an exclusive public sector domain in South Asia with reluctance of health ministries to publicly acknowledge the role of private providers and share resources for expanding access to primary care. Nonetheless, the present policy environment comprising of well‐established inpatient‐based national health insurance initiatives in India, Pakistan and unfolding insurance‐based pilots in Bangladesh, provides an opportune window for links with PPPs for primary care.

**BOX 1 Recognition of private sector engagement in national health policies**

1

- Bangladesh's National Health Policy of 2011 recognises building partnerships with private organizations and the PPP Act of 2015 provides the groundwork for the Ministry of Health and Family Welfare (MoHFW) for integrating private clinics into the provision of public health service.
- India's National Health Policy of 2017 explicitly refers to strategic purchasing of private provider services with an emphasis on hospital services as well as PHC areas of disease control in areas of geographical gaps.
- Pakistan's National Health Vision Pakistan 2016–2025 sets out private sector engagement as one of the pathways towards achieving National SDG targets, and PPP Acts formulated in different provinces provide the legal basis for use of PPPs to expand access to quality care (reference national health vision Pakistan).

### 2.2 Local Needs Shaping a Bottom‐Up Agenda

PPPs for primary care have been led by sub‐national governments in response to needs of local constituencies. Filling geographical gaps in health coverage has been a major underlying need resulting in diverse manifestations of PPPs for PHC. Examples include complementing sparse government PHC infrastructure in cities with private providers (Bangladesh, India) [16, 17], supplementing demand for maternity services in rural areas (Bangladesh, India) [18, 19], and mobile clinics support to government PHCcentres in remote areas (India) [20]. Improving the functioning of government PHC centres has been another major need for PPPs with examples ranging from the introduction of diagnostic services within government PHC centres (India) [21] to providing management control of poorly performing PHC centres to private partners (India, Pakistan) [22, 23] In fewer instances, government managed PPPs have been deployed to meet disease control targets prevention usually instigated by donor agencies, implemented with donor co‐financing. Prominent examples involve PPPs for TB control [24], nutrition [25], HIV prevention [26]but have mostly failed to be sustained after termination of donor co‐financing The exception is the Patient Provider Support Agency (PPSA) initiative for TB control in India based on a locally engineered process that started with a small donor funded pilot and expanded to state and national levels with progressive government leadership [24]. India has the most diverse mix of PPP modalities, Bangladesh has delivered a large PPP for urban PHC with smaller instances of maternity care PPPs, whereas Pakistan has largely focused on management contracts with private sector to revive state‐owned primary care facilities (Table 1).

**TABLE 1:** PPPs for primary care services: Initiatives, scale and beneficiaries.

PPP area	Countries	Initiatives	Geographical scale	Beneficiaries
Urban healthcare	Bangladesh	New initiative under discussion urban primary health Care:1998–2024 [18]	12 city corporations and 14 municipalities	Residents of low‐income urban localities, target to reach 30% of poverty scorecard holders
	India	Mohalla (community) clinics (hybrid model) in Delhi, with extension to Punjab: 2015‐ongoing [21]	Low‐income community localities	Low income/disadvantaged localities of 10,000–15,000 population
Management of government PHC centres	India	Management of PHC centres in several states: 1996‐ongoing [26]	Remote hilly/rural areas in Karnataka, Assam, Arunachal Pradesh, odisha, other states	Rural/tribal population in the defined vicinity of identified PHC centres
	Pakistan	Several examples in two of four provinces of Pakistan	Sindh province, 132 rural health centres, 19 BHUs/dispensaries, several districts	Rural population residing in catchment of health facilities
		KP new initiative:		
		Sindh public‐private partnership initiative 2017–2021 [28]	Cluster of health facilities in 6 crisis hit districts	
		Revitalising health services, khyber pakhtunkhwa: 2012–15 [29]		
		Provincial adaptations of national PPHI: 2013‐ ongoing	Government basic health units in selected provinces	Rural population residing in catchment of health facilities
		Presidents primary care initiative sindh		
		Chief Minister's primary care initiative Punjab		
		President's primary healthcare initiative: 2008–2012 [30]	2393 basic health units over 82 districts nationally	Rural population residing in catchment of health facilities
		Contracting of health facilities under district devolution: 2001–07 [31]	Selected poorly performing health facilities in occasional districts	Disadvantaged localities within rural districts
Additional services to existing government PHC centres	India	Vaidya pariksha scheme	Across the province of Andhra Pradesh	Rural population requiring diagnostic services in public facilities within vicinity of the community
		2016–2020 (free diagnostics) [24]		
	India	Mobile clinics and its variations	Various states (e.g., Uttarakhand, Assam, Bihar, odisha, Madhya Pradesh)	Populations living in far flung rural, hilly areas, river islands. Free for households with poverty cards
		2009–15 [25]		
Maternity services	Bangladesh	Maternal health voucher scheme: 2006–23 [19]	Successive expansion to 53 sub‐districts	Low‐income pregnant women holding poverty scorecard
	India	Chiranjeevi Yojana,2005–2023 [23]	All 25 districts of Gujarat state	Low‐income women holding poverty‐cards or any pregnant women approved by local public authority
Disease screening and services	India	Pilot upscaled to districts and then national level	Initial 3 district pilots scaled up to 385 districts across several states: countrywide expansion since 2019	TB patients within catchment area of identified geographical area
		Private provider partnerships for TB, 2013‐ongoing [22]		
	Pakistan	Federally led national initiative: NGO contracting for HIV services: 2003–2008 [32]	12 major cities	Four high risk groups
	Bangladesh	Federally led national initiative:	Of 105 thanas, some contracted out to NGOs, in others government implemented with limited support of NGOs	< 2 years children in rural populations
		Bangladesh integrated nutrition project—main component: community‐based nutrition 1995–2002 [20]		

Notably PPPs in Bangladesh, India and Pakistan have been placed in remote rural areas and urban slums driven by an implicit pro‐poor agenda. Some PPPs have even leveraged on national poverty scorecard schemes to better target low‐income groups ‐ examples include maternity schemes across Bangladesh (MHV) [18], India's Gujarat (Chiranjeevi) [19] and mobile clinics in India [20] (Table 1). While PPPs have served disadvantaged areas, the extent of utilisation by the poorest in these areas is uncertain and insufficiently assessed. Strengthening mechanisms to ensure equitable uptake must feature more centrally within the design of future PPP arrangements [27, 28, 29].

## 3 Modalities of Home‐Grown PPPs

PPP modalities in Bangladesh, India and Pakistan can be characterised by contracted services, private partners and payment modalities. There are noticeable common features across the three countries.

### 3.1 Contracted Services

PPPs for primary care that are fully financed by country governments have focused on general primary care services (Table 2). The services are often loosely defined with priorities left to private partners and local government stakeholders to interpret during implementation. Well‐defined primary care services are seen more recently and examples include management contracts in Pakistan's Sindh and Khyber Pakhtunkhwa provinces based on Essential Health Service Package, contracts for laboratory services in several Indian states and Mohalla clinics in Delhi [17, 21, 30, 31]. Conversely, PPPs co‐financed by international donor agencies are seen to have tightly defined primary care services and monitoring targets and essentially rely on market capability to deliver to written specifications as opposed to the adaptation, iteration process of defining services as seen in solely government financed contracts (Table 2). Available evidence shows that PPP initiatives show promising service volumes for general out‐patient (OPD) visits, female OPD visits, maternity services and laboratory tests, however performance is less promising for preventive care services unless these are specifically made the focus of the contract [27, 28, 29, 30, 31, 32, 33, 34, 43]. PPPs are also documented to result in cleaner well‐maintained facilities, longer opening hours, better presence of female staff, medical supplies availability and patient satisfaction but less is known about the technical quality of care within PPP arrangements. Increase in population‐based coverage is reported for some but not all PPPs [29, 31, 34] and difficult to ascertain due to either absence of baseline measurements or confinement of PPP arrangements to facility‐based health services with authority for outreach services often retained by government counterparts.

**TABLE 2:** PPP for primary care services: Services, modalities, private partners.

PPP area	Countries	Services	Modalities	Partners
Urban healthcare	Bangladesh: Urban primary health care: Dhaka, cities	Essential service package for PHC (free services)	i) Co‐financing by local government + ADB; ii) deliverable based payments linked to itemised budgets; iii) contracts with NGOs to establish network of PHC centres, iv) competitive selection of partners	24 national NGOs
	India:	OPD consultation and medicines (free services)	i) State government's financing, use of special initiatives funds for rented clinics premises for clinics and operational costs, ii) contracts with private doctors to provide consultations at fixed fee per patient	Private general practitioners in government designated clinics
	Mohalla clinics, Delhi, Punjab			
Management of government PHC centres	India: management contracts in Karnataka, other states	Minimum of basic service package for primary health centres (free services)	i) State government financing, use of recurrent funds; ii) global budget; iii) partnership agreement for management of government PHC centres in remote areas	NGOs, medical universities
	Pakistan: Market based contracts, sindh, khyber pakhtunkhwa	Essential health service package (EHSP) for PHC (free services)	i) Sindh: State government financing with recurrent funds,	NGOs, medical charities, consulting firms
			i) KP: State government financing with development funds/donor co‐financing	
			ii) Global budget with deliverables; iii) contracts for management of government PHC centres, iv) competitive selection of private partners	
	Provincial adaptations of national PPHI:	General PHC services, loosely defined (free services)	i) State government financing, use of recurrent funds ii) global budget; iii) contracts for management of government PHC centres, iv) sole selection of NGO/company	State funded purpose‐built autonomous PHC company
	Sindh, Punjab			
	President's primary healthcare initiative (PPHI)	General PHC services, loosely defined (free services)		State funded NGO with ministry of industry
	Specific health facilities under district devolution	Varying PHC services, loosely specified	i) District government financing, use of recurrent funds ii) global budget; iii) contracts for management of government PHC centres, iv) sole selection of NGO/company	National NGOs, affiliates of INGOs
Additional services to existing government PHC centres	India:	Identified diagnostic/laboratory tests: sample collection, transportation, testing, submission of test results (free services)	i) State government financing use of recurrent funds, ii) fixed per patient tariff, iii) contracts for provision of laboratory services to government PHC centres; iv) sole selection of partner	Private medical agency
	Free diagnostics at government facilities			
	India: Mobile clinics and its variations	Defined package of PHC services, and basic diagnostics through mobile vans (subsidised fee)	i) State government recurrent financing, ii) payment for private partner operational costs + government provision of supplies/medicines, iii) contract with private organizations, iv) competitive selection of partners.	Uttarakhand: 4 private providers (UMHRC); arogya rath: 3 private providers
				Madhya Pradesh: 13 private providers
Maternity services	Bangladesh: Maternal health voucher scheme	ANC delivery, emergency referral, PNC, transport	i) Government financing, transport stipends; ii) partnership agreements with private facilities through a governmevt Vouchmr Managemeat Agency; iii) vouchers exchanged at eligible centres for maternity services	Private health facilities medical charities + government health facilities
	India:	ANC, delivery, assisted birth, C‐sections, nutrition, transport, newborn care if required (co‐payment)	i) State government financing, use of recurrent funds, ii) pre‐negotiated rate per 100 deliveries conducted; iii) contracts with empanelled private providers for maternity services	Partnerships with 572 private providers across the province
	Maternity services, chiranjeevee recently integrated into national health insurance			
Disease screening and services	India:	TB screening, diagnosis and DOTs treatment (free services)	i) Transitioned from donor pilots to federal government + donor co‐financing; ii) payment linked to bundled or specific services; iii) performance‐based contracts with locally adaptive services; iv) competitive selection of intermediary private providers	NGOs as intermediary partners enroled 70,146 private clinics 34,105 hospitals and 9710 laboratories
	Private provider partnerships for TB			
	Pakistan: HIV prevention services	Defined package of HIV prevention and care services (free services)	i) National/state government co‐financing + world bank support, use of development funds, ii) deliverable‐based contracts; iii) competitive selection	Private firms/large NGOs partnered with smaller NGOs
	Bangladesh: Integrated nutrition project	Nutrition screening, counselling, supplementary feeding (free services)	National government development funds + World bank financing; ii) partnership agreement with NGOs to deliver nutrition services, iii) priced deliverable based contracts; iv) competitive selection of partners	NGOs delivered services in defined geographies

### 3.2 Partners

Private partners of government managed PPPs for PHC are notably local organizations (Table 2), Noticeably, locally based private partners have diversified over time from earlier PPPs with selected charities to engagement with NGOs, university hospitals, private commercial organizations, private clinics, hospital, consulting agencies and large healthcare networks (Table 2). This signals signal availability of a reasonable domestic private healthcare market in South Asia to implement forward.

International NGOs have rarely participated in government managed PPPs usually due to preference for direct financing by donor agencies. A positive trend is also seen of many PPPs moving from sole source selection of private partner to competitive selection of partners intended to draw in those with better proposals. However, there has been questionable success in attracting the best equipped private providers. For example, the PPSA for TB in India had weak competition from private sector due to underpriced contracts [24]^,^. Similarly, PPPs for PHC management contracts in Pakistan even when competitively tendered have usually drawn in few applicants [32, 33]. Similarly, better equipped private providers did not participate in maternity schemes in Bangladesh and India due to pricing and trust issues [36, 37] (Table 3). Even new national programs, such as the India's PPPs for TB continue to be affected by communication and trust gaps between private and public sectors [44]. Insights from PPP implementation process indicate that private providers apprehension of delayed release of payments and cumbersome administrative processes reduced interests in PPPs whereas availability of communication and troubleshooting support were positively perceived for PPP engagement^,^ [32, 33, 38, 39].

**TABLE 3:** PPPs for primary care services: Enablers and barriers.

PPP area	Countries	Enablers	Barriers
Urban healthcare	Bangladesh: Urban primary health care: Dhaka, cities	Aligned with needs of local constituency Championing + administrative support from local bodies ministry	Actual delivery costs higher than costed priced in contracts Donor procurement rules contributed to administrative + payment delay Lack of municipalities role in monitoring leading to turf issues Weak integration with the MoH's PHC reform initiatives
	India: Mohalla clinics, Delhi, Punjab	Aligned with needs of local constituency High policy ownership Timely replenishment of medicines/supplies	Lack of policy, legal and organizational oversight framework. Absence of quality framework for monitoring private providers Lack of system to validate private provider reimbursement claims
Management of government PHC centres	India: management contracts in Karnataka, other states	Aligned with needs of local constituency High ownership by state government Simple model, easy to implement Recurrent sustainable financing Historical experience from other states	NGO ‐government relationship varied in line shifts in local leadership Turf fights on resources at lower‐levels bureaucracy Staff resistance for shift to private management Payment delays Less effort on community outreach, vague scope of services
	Pakistan: Second generation PHC management contracts, sindh, khyber pakhtunkhwa	Sindh + KP: Aligned with needs of local constituency Strong ownership + administrative facilitation by political‐bureaucratic coalitionHistorical experience of prior initiatives Assistance from finance for legal framework Global budgets + recurrent financing allowed speedy staff/medicine deployment/refurbishments/innovations	Sindh + KP: Turf issues with district health authorities Staff resistance for shift to private management Weak competition for contracts Weak enforcement of quality leadership and standards Disconnects with other PHC national initiatives and planning
			Sindh: Under‐priced to implement essential health service package, cash flow issues from heavy volumes demotivated private providers
			KP: Use of development funds and tight donor rules led to onerous process, delays in fund releases, loss of interest from private partners
	First generation PHC management contracts i) provincial adaptations of national PPHI: Sindh, Punjab ii) National President's primary healthcare initiative (PPHI)	Aligned with needs of local constituency Strong ownership + administrative support from politicians and higher bureaucracy Historical experience of prior initiatives Flexible budgetary space allowed speedy staff/medicine deployment, refurbishments	Lack of oversight links to health ministry/health departments Turf issues with district health authorities Resistance by government staff to be managed by NGO Cherry picking services, less emphasis on preventive care
	Precursors of management contracts: Specific health facilities under district devolution	Local ownership and imperative to improve services Decision making and budgetary allocation authority with local governments	Vaguely specified services, non‐standardized delivery Weak capacity to monitor, relied on trust, relational working
Additional services to existing government PHC centres	India: Free diagnostics at government facilities	Aligned with needs of local constituency High ownership by state government Simple model, easy to implement	Change of government led to closure of scheme Delayed release of payments Lack of speedy monitoring systems for claim verification Cost containment difficult due to per patient tariffs
	India: Mobile clinics and its variations	Aligned with needs of local constituency High ownership by state governments Simple model easy to implement Historical experience from other states	Uneven non‐standardised services Weak capacity to monitor quality of services and validate volumes
Maternity services	Bangladesh: Maternal health voucher scheme	Sufficient additional funding provided to facilities Initial perseverance helped overcome teething issues Knock‐on effect on public sector facilities to provide better quality services in competition with private providers	Complex to administer requires a parallel administrative mechanism More efficient funding modalities required Quality assurance mechanisms absent private sector participation less than expected
	India: Maternity services, chiranjeevee Recently integrated into national health insurance	Aligned with needs of local constituency High ownership +a administrative facilitation by local government Involvement of gynaecologist association helped mobilise local private providers. Simple model to implement	Support tied to individuals: weak buy‐in after leadership change Lesser equipped private providers participated Better equipped private providers found tariffs to be too low for quality of service required/feared misuse of funds Bureaucratic procedures led to withdrawal of private providers Lower tariffs under insurance integration further reduced interest of private providers
Disease screening and services	India: Private provider partnerships for TB	Adaptation from pilot, to larger project, to national scale up sensitisation and conversion of local, state, national leadership National government financing with sharing of authority and resources with sub‐national government	Not all states willing to adopt. Bureaucratisation of process after transition to government delayed payments with excessive verifications, insufficient IT reporting capacity Low interest of private sector, limited number of NGOs applied as PPSA
	Pakistan: HIV prevention services	Favourable environment for HIV intervention Donor support and advocacy Availability of NGOs with credible work experience	Weak provincial government buy‐in Bureaucratised, tight donor shaped result in delays, fatigue and capacity issues in government and NGO partners Projectized time‐bound funding
	Bangladesh: Integrated nutrition project	Donor support and advocacy Learnings from other country projects	Heavy resource requirements for NGO contracts Translation into nutrition practice impeded by resource and time constraints faced by mothers Projectized time bound funding

### 3.3 Funding and Payment Arrangements

PPP arrangements that are fully financed by country governments are more commonly seen in India and Pakistan. These arrangements use less detailed contracts and simple payment modalities in line with government capacity to disburse and monitor. Payments to private partners are commonly made through global budgets for general primary care services and are popular both for cost‐containment as well as simplified administrative implementation. Negotiated tariffs have been used by PPPs for purchase of selected services such as in the case of maternity care PPPs. Selective itemised funding with additional support from government provided medical supplies is another popular mechanism seen in government financed PPP contracts.

PPP initiatives that are only partially financed by country government are more commonly seen in Bangladesh, with fewer instances in India and Pakistan. Co‐financing has usually been provided by multilateral development banks. These PPP initiatives are noticeably under‐written by the multi‐lateral agency's procurement and financial rules, typically involving multiple payments milestones tied to reporting deliverables and more elaborate processes of payment releases (Table 2). Implementation experiences are discussed further under enablers and barriers.

## 4 Enablers and Constraints Within the PPP Ecosystem

### 4.1 Power Location Within Government

Power to introduce PPPs in South Asia has usually rested with political executives, civil servants and local governments responding to needs of constituencies. Hence, the management of PPPs for primary care has been housed in government entities outside of the Ministry of Health or PPP units within health departments that report vertically to the finance or planning ministries. Bangladesh's large PPP for urban primary care was led by the local government closely linked to the agenda of well‐being for urban constituencies. India's Mohalla clinics, Chiranjeevi maternity scheme and Karnataka management contracts were managed by municipal, district, or provincial chief minister's special initiatives rather thahealth departments. At times PPPs have even been deliberately housed outside the ministry of health to protect from turf fights by health departments. For example, Pakistan's large‐scale national President's Primary Healthcare Initiative for management of rural government primary care centres was housed in a specially created unit in the Ministry of Industry to protect it from resistance put up by health departments on transfer of public sector budgetary resources to private providers [38]. Although the location of PPP management outside health ministries has protected PPPs from turf wars but at the same time, it has created fundamental disconnects with national primary care targets and UHC reforms [35, 36, 38] (Table 3). Policy, legislative and risk management frameworks to support PPPs for health service delivery remain undeveloped or at best borrowed from PPP infrastructure frameworks of finance ministries that are not adapted to the healthcare context. Furthermore, there has been little effort across all three countries to develop private sector engagement roadmaps for the health sector under which a direction for PPPs could be provided.

### 4.2 Championing and Stakeholder Coalitions

PPPs for primary care in Bangladesh, India and Pakistan have relied on championing from senior civil servants, and trusted public figures to over‐ride resistance and mistrust from government health staff (Table 3). Nonetheless, PPP arrangements that have been heavily dependent on individuals have run into risks of discontinuation once the ‘champions’ no longer exerted influence. For example the Chiranjeevee scheme in India lost momentum after the transfer of the Health Commissioner. Similarly, the PPP for laboratory services in Andhra Pradesh was discontinued after a change in t government [13, 15]. Better sustainability of PPPs is seen when coalitions of stakeholders have come together as seen for management contracts in Pakistan where politicians and the civil bureaucracy have maintained a tight coalition in persevering with management contracts for government PHC clinics. Championing and coalitions beyond the health sector have proved instrumental in overcoming mistrust of health ministry staff reducing administrative delays, building fiscal space in budgets and negotiating with health workforce resisting working under private sector management [22, 26, 40, 44] (Table 3).

### 4.3 Role of Recurrent Versus Development Funding

Type of financing provided for PPPs in Bangladesh, India and Pakistan, is seen to be linked both with the timely release of funds as well as the continuity of PPPs. Most PPPs for primary care are financed from recurrent funding and supplemented by special initiative funds (Table 2). PPPs funded at the outset from public sector recurrent budgets have experienced lesser delays in payment releases to private partners and faced lesser disruptions. Conversely, PPPs supported with donor co‐financing draw on government development funds that are tied to a time‐bound project cycle and frequently face payments delays and are more vulnerable to budgetary cuts [39, 40, 41, 42]Reliance on projectized development funding creates uncertainty about successful transition of popular PPP arrangements as seen for example in the case of the urban primary care initiative in Bangladesh [39] or quite often led to discontinuation as in the case of HIV control and PHC revitalisation contracts in Pakistan [33, 42] (Table 3).

### 4.4 Government Capacity

Sub‐national governments in India, Pakistan and Bangladesh have relied on diffusion of experience to establish PPPs and often replicated simple local designs that have worked elsewhere in the country. Prominent examples include Mohall clinics upscaled from Delhi to Punjab, a, PPPs for mobile clinics across Indian states of Uttarakhand, Assam, Bihar, Odisha, Madhya Pradesh, expansion Bangladesh's urban health programme and a generation of PPPs for managing PHC health facilities across Pakistan's provinces, amongst other examples (Table 1).

Government capacity to manage PPPs remains weak in all three countries. Although there are established government procurement systems for supplies and infrastructure but dedicated units for health PPPs are not usually seen within health ministries. PPP units have been established by few sub‐national governments that have considerably advanced PPPs. However, these are constrained by contract writing, risk management and pricing skills. PPPs co‐financed with international donors are run by donor‐supported project management units (PMUs) that remain siloed from the institutional architecture of health ministries and create further fragmentation. Capacity built within PMUs usually gets dissipated on project closure.

Monitoring of PPPs continues to rely largely on paper‐based monitoring systems which has slows down the verification of service delivery This also creates payments delays for PPPs in cases where governments have tied to establish some link to proof of delivery. Recent introduction of digital reporting of volumes has been a game changer in performance monitoring of PPPs. Examples include the TB PPSA in India and digitized balanced score card‐based health facility assessments in Pakistan. Digitized performance reporting has helped validate work, reduce suspicion of under‐performance and helped in timely release of payments to private providers in TB PPSA in India [24]. However quality oversight and standardisation of services remains one of the missed opportunities within all PPPs for primary care. In recent years, healthcare quality standards have been established for enforcement by semi‐autonomous healthcare commissions in Pakistan, by regulatory authorities for hospitals and laboratories in India, and the health services directorate in Bangladesh. However there has been little attempt to build links with the national regulatory authorities and PPPs management for the pre‐qualification of private partners and monitoring of minimal accepted quality guidelines during implementation. Capacity and resources for quality enforcement are also weak.

### 4.5 Accountability and Bureaucratisation

PPPs in India, Pakistan and Bangladesh have been constrained by either too few explicit controls or excessive bureaucratisation of contract management. PPPs fully funded by country governments have often relied on simple contracts for primary care services supported with global budgets. However, these do not provide adequate financial controls over PPPs and create risks of financial non‐compliance, but have provided operational ease for both the public and private sector. Global budgets have helped in speedy roll‐out of services by providing financial flexibility to private partners for spending on staff recruitment, supplies and refurbishments [13, 22]. Moreover, provision of decision‐making space to private providers has been important for introducing innovations such as digital reporting, digitized staff attendance, supplies monitoring and telemedicine consultations for service functionality [32, 39].

Donor co‐financed PPPs in South Asia have detailed service milestones and payments. These are underwritten by donor rules of business and although intended to improve accountability have been administratively cumbersome for both private and public sector partners. Extensive paperwork and reporting are required and seen to be mismatched with local government and private market capacities. For example heavy administrative processes resulted in administrative delays [39, 42] as well as loss of interest and early exit of private providers [33, 37] (Table 3). Both private providers and government stakeholders across Bangladesh, India and Pakistan are commonly wary of extensive bureaucratisation of the PPP process. Close coordination and trust have served as informal but important controls helping governments and private partners to monitor implementation, troubleshoot administrative issues and overcome vested interference (Table 3).

## 5 Conclusion

The momentum of PPPs for primary care in South Asian countries is locally driven, substantial in scale, but uncoordinated and must be integrated into systematic UHC planning. Bangladesh, India and Pakistan have different trajectories of PPPs, but common pathways, drivers and constraints. Local needs have been the starting points for PPPs with leadership by sub‐national governments and reliance on diffusion of practice across states. Common interests of local constituencies and shared bureaucratic coalitions across states have facilitated adoption of PPP innovations (Table 4). Upscaling and growth into national initiatives in South Asia has essentially relied on a bottom‐up process of ideation and adaptation rather than centrally designed initiatives.

**TABLE 4:** PPPs for Primary care: Summary of Enablers and Barriers.

Ideation and pathways			
Critical enablers		Critical barriers	
Prior experience, ideation & adaption over time, diffusion of practices	Political‐bureaucratic coalitions beyond health sector	Centralised top‐down initiatives weakly adopted and sustained, even if well designed to public health needs	Turf fights at lower levels of government, resistance by health staff require persevered political‐administrative support
Locally identified needs, resonating with local constituencies		Several disparate PPP initiatives, lack of strategic national direction	Fire‐walled implementation leads to lack of links with PHC planning service guidelines and targets; with UHC planning
		Erosion of experiential PPP lessons due to absence formalised stewardship structures	

Modalities		Private sector participation	
Critical enablers	Critical barriers	Critical enablers	Critical barriers
Simple contracting and payment process	Bureaucratised processes and projectized funding cause delays, administrative fatigue excessive time investment	Participation of local private sector	Weak private sector competition: concerns of pricing, bureaucratised engagement with the government, timely payments
Recurrent financing	Weak exercise of quality leadership, risk of underperformance	Increased use of competitive contracting of private providers	
Decision space for innovations	Weak pricing skills resulting into under‐costing of global budgets or cost containment issues with patient tariffs	Range of private sector seen	
Digital monitoring where introduced helps with performance monitoring and claim validation	Digital monitoring not a norm in most PPPs		

Key drivers shape the initiation, implementation and continuation of PPPs (Table 4). Administrative‐political support beyond the health sector is seen to be instrumental in both the instigation of PPPs as well as protection from turf wars. Use of recurrent public funds for PPPs is linked to a smother process of payment disbursement and better continuity of funding whereas projectized funding from development budgets exacerbates delays. Simplified payment systems, decision space for innovative responses and speedy rollout by private partners as well as relational management to troubleshoot are seen to be instrumental in successful steering of PPPs in South Asia rather than legal transactional controls.

Common constraints are seen in all three countries weakening PPP impact and coherence with national UHC goals (Table 4). First, PPP contract design either features too little accountability thereby heightening risks for the public sector or there is excessive accountability driven by donor rules of business that are mismatched with country government and NGO capacity. Second, private sector concerns of trust, imaging and pricing are not meaningfully addressed in designing of partnerships. Third, there is weak performance monitoring and risk management capacity within governments, and capacity is further compounded by institutional erosion of experiential lessons. Finally, the fire‐walled implementation of PPPs disconnects them from national primary care planning targets, financing and quality regulation under UHC.

In conclusion, we provided a process‐based understanding of PPPs for PHC emphasising attention to pathways and drivers to advance PPPs for UHC. Emphasis must be on local simplified ideation, progressive adaptation and allowing contextual diversity under a larger UHC planning architecture, rather than centralised sophisticated one‐fit designs. Success drivers of PPPs from the South Asian context must be paced at the centrefold of PPP design and include political‐bureaucratic support beyond the health sector, simplified contractual and payment systems to provide operational ease and decision space, and the use of relational management and digital monitoring for resolving issues. Our paper also argues for a shift in international donor assistance from funding projectized short‐lived PPPs funding to longer‐term investment in technical assistance for building public sector capacities in stewardship, private sector engagement skills as well as the more traditional performance management capacity. Future research on PPPs should probe how to further diversify from NGO partnerships to engaging and managing private commercial providers for public sector goals.

## Author Contributions

S.Z. framed the article with A.V.R. and M.E.C.. S.Z., A.V.R., M.E.C., F.A., P.B. collated data and draughted the synthesis. All authors read and approved the final paper for submission.

## Ethics Statement

The authors have nothing to report.

## Conflicts of Interest

Authors confirm that there are no competing interests.

## Data Availability

The data that support the findings of this study are available from the corresponding author upon reasonable request.
